# Pore Structures for High-Throughput Nanopore Devices

**DOI:** 10.3390/mi11100893

**Published:** 2020-09-26

**Authors:** Sou Ryuzaki, Rintaro Matsuda, Masateru Taniguchi

**Affiliations:** 1 Institute for Materials Chemistry and Engineering, Kyushu University, Fukuoka 819-0395, Japan; matsuda@ms.ifoc.kyushu-u.ac.jp; 2 PRESTO, Japan Science and Technology Agency (JST), Saitama 332-0012, Japan; 3 The Institute of Scientific and Industrial Research, Osaka University, Osaka 567-0047, Japan

**Keywords:** nanopore, high-throughput, electric field, nanofluidics

## Abstract

Nanopore devices are expected to advance the next-generation of nanobiodevices because of their strong sensing and analyzing capabilities for single molecules and bioparticles. However, the device throughputs are not sufficiently high. Although analytes pass through a nanopore by electrophoresis, the electric field gradient is localized inside and around a nanopore structure. Thus, analytes located far from a nanopore cannot be driven by electrophoresis. Here, we report nanopore structures for high-throughput sensing, namely, inverted pyramid (IP)-shaped nanopore structures. Silicon-based IP-shaped nanopore structures create a homogeneous electric field gradient within a nanopore device, indicating that most of the analytes can pass through a nanopore by electrophoresis, even though the analytes are suspended far from the nanopore entrance. In addition, the nanostructures can be fabricated only by photolithography. The present study suggests a high potential for inverted pyramid shapes to serve as nanopore devices for high-throughput sensing.

## 1. Introduction

One of the most extreme nanofluidics in nanobiotechnology is a channel with the size of a single molecule, because these nanochannels enable us to handle and analyze single molecules. For physiological tissues, nanochannels, e.g., membrane transport protein, are also essential systems for the detection and discrimination of single biomolecules in our bodies [1]. Nanopore devices have been a research focus from the nanochannel perspective because the devices are intended to mimic the functions of membrane transport proteins, although these devices are only available for large molecules or bioparticles at the moment [2,3,4], e.g., DNA sequencers [5] and single-bioparticle analyzers [6].

**Figure 1:**
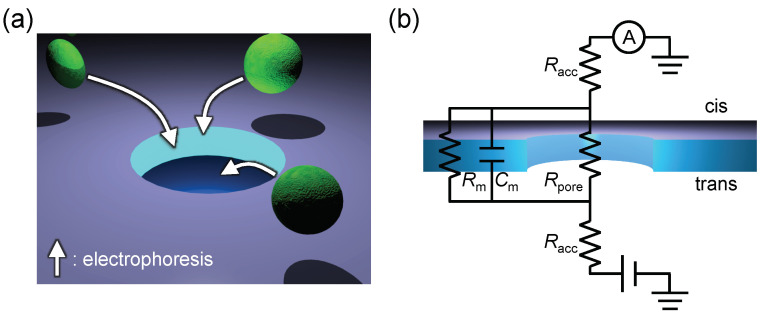
Schematic illustrations of a nanopore device. (**a**) Schematic illustration of analyte translocations by electrophoresis around a nanopore. As the potential drop is localized inside and around a nanopore, only analytes around the nanopore entrance can be detected. (**b**) Equivalent circuit of a nanopore device. *R*_ass_, *R*_pore_, *R*_m_, and *C*_m_ correspond to the access resistance, the pore resistance, the membrane resistance, and the membrane capacitance, respectively.

**Figure A1:**
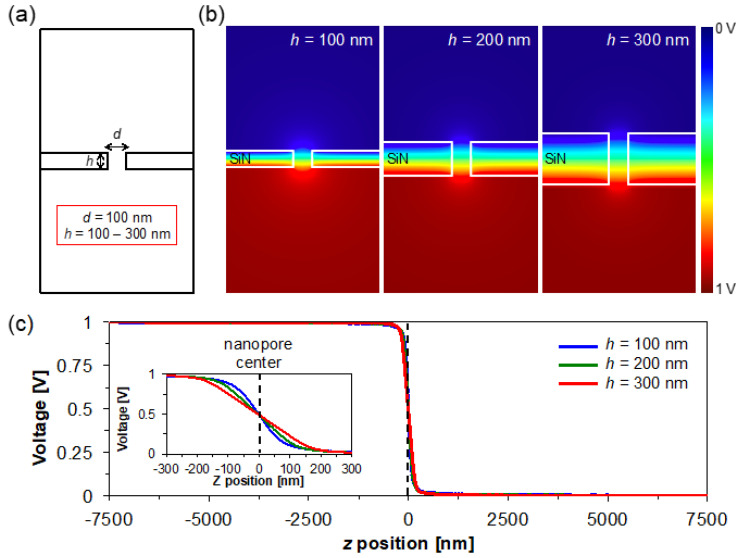
Simulation results for the conventional cylindrical nanopore structures. (**a**) Schematic illustration of the simulation model. (**b**) Cross-sectional potential distributions at *y* = 0 for the nanopores with *h* = 100 nm, 200 nm, and 300 nm. (**c**) Potential dependences on the *z* position on the center axis (*x* = 0, *y* = 0) for the nanopores with *h* = 100 nm (blue), 200 nm (green), and 300 nm (red), and enlarged view near *z* = 0 (inset). The potential drops are respectively 0.55 V, 0.69 V, and 0.78 V inside the nanopores with *h* = 100 nm, 200 nm, and 300 nm. The full electric field gradients of 0–1.0 V exist within *z* = −200–200 nm in the present structures (Figure A1c), meaning that analytes suspended more than 200 nm above the nanopore center (*z* = 0) cannot pass through a nanopore by electrophoresis unless analytes at a distance greater than the capture radius diffuse into the region incidentally by Brownian motion. These results are comparable to previous reports about the conventional cylindrical nanopore structures [29].

A nanopore device typically consists of an insulating membrane with a single nanopore structure, which physically separates the cis and trans chambers, and it has an electrode pair across the membrane. The chambers are filled with an electrolyte solution, and analytes dispersed in the cis chamber translocate to the trans chamber via a nanopore by electrophoresis (Figure 1a). Nanopore devices provide size and/or shape information for single analytes passing through a nanopore by probing temporal changes in the ionic current because an analyte excludes ions inside a nanopore during the translocation and causes an ionic current blockade with amplitudes proportional to its feature size [7,8]. Nanopore structures thus enable label-free sensing of single biomolecules and bioparticles by estimating the size and/or shape of the analytes passing through a nanopore from the pulsed ionic current blockades. However, the device throughput is not sufficiently high because the potential drop is localized inside and around a nanopore structure (Figure A1). Thus, it is difficult to drive analytes far from a nanopore with electrophoresis, meaning that only analytes around the nanopore entrance can be detected unless analytes at a distance greater than the capture radius diffuse into the region incidentally. A large number of publications report how to improve the throughput by optimizing electrolyte concentration [9], controlling the local charge on nanopore walls [10], and increasing the number of nanopore structures in a device, such as an array structure [11,12,13]. However, the throughput has only increased several times per nanopore structure because the potential drop is basically localized inside and around a nanopore structure. One of the relevant factors for the localized potential drop is a higher pore resistance (*R*_pore_) compared with access resistances (*R*_acc_), which corresponds to the ionic current resistances inside a nanopore and between the electrode and the nanopore, respectively. Properties of nanopore devices are often discussed with a simple equivalent circuit consisting of the *R*_pore_ and the *R*_acc_ in a series (Figure 1b). In this instance, each resistance can be described by

(1) $$R_{\mathrm{pore}}=\frac{\alpha h}{\pi {\left(D_{c}/2\right)}^{2}}$$

(2) $$R_{\mathrm{acc}}=\frac{\alpha }{D_{c}}$$

where *α* is the resistivity of the buffer solution, *h* is the thickness of a nanopore, and *D*_c_ is the diameter of a nanopore [7,14]. As *R*_pore_ and *R*_acc_ are in a series, a higher *R*_pore_ compared to *R*_acc_ causes more drastic potential drop inside a nanopore. Although a larger *D*_c_ can make the ratio of *R*_pore_ to *R*_acc_ smaller, a larger *D*_c_ results in a lower signal/noise (S/N) ratio of the ionic current blockades due to analyte translocations. The insulating membrane is also the factor causing the localized potential drop because the permittivity of the insulating membrane is a relatively small value, and the combined resistance of *R*_pore_ and *R*_m_, which is (*R*_m_ × *R*_pore_)/(*R*_m_ *+ R*_pore_), is considerably higher than *R*_acc_. Here, *R*_m_ corresponds to a resistance of the insulating membrane. Therefore, the potential shows a drastic drop inside the membrane and nanopore, resulting in almost flat electric field gradients in the cis and trans chambers, and small gradients in the vicinity of the nanopore entrance and exit (Figure A1). Although homogeneous electric field gradients in each chamber are obtained using a conducting membrane, the noise caused by the membrane in the ionic current increases because of the high capacitance of a conducting membrane (*C*_m_) [15].

**Figure 2:**
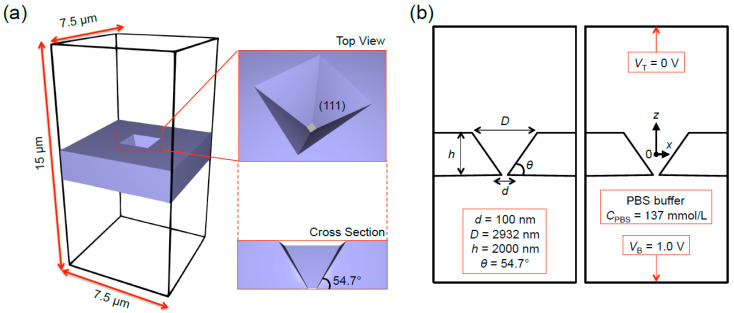
Simulation model for the inverted pyramid (IP)-shaped nanopore devices. (**a**) The three-dimensional IP-shaped nanopore model with *D* = 2932 nm and *d* = 100 nm. The *xy*-size and height for the cis and trans chambers are 7.5 × 7.5 μm and 6.5 μm, respectively. As the IP-shape nanopore is envisioned to be fabricated by an anisotropic wet etching on a (100) silicon wafer, the slope angle is 54.7°. (**b**) Definitions of the model size, the coordinate origin, the bias voltage, and the concentration of phosphate-buffered saline (PBS) buffer.

Here, we report inverted pyramid (IP)-shaped nanopore structures consisting of a layered structure of insulating silicon nitride (Si_3_N_4_) and semiconducting Silicon (Si) membranes to even out the electric field in a nanopore device. This structure is expected to cause a homogeneous potential drop between the electrodes and the nanopore structure because the large pore entrance of the IP shape (*D*) causes a smaller *R*_pore_ than that of a normal cylindrical nanopore with *D*_c_ = *d*. Here, the side lengths of the top and bottom pores of the IP-shaped nanopore are respectively defined as *D* and *d* (Figure 2). In addition, the optimal conductivity and permittivity of the Si membrane are discussed from the viewpoint of both the throughput and the S/N ratio of the ionic current blockade. In this study, the electric field gradients in nanopore devices are examined using a multiphysics model simulation constructed from hydromechanics, electromagnetics, and ionic transport theory [16,17], and we also discuss the availability and a fabrication method for the IP-shaped nanopore from the perspective of high-throughput nanopore devices.

## 2. Methods

To begin, we established a multiphysical model to evaluate the electric field gradients. The model consists of three physical equations (Poisson–Boltzmann, Nernst–Plank, and Navier–Stokes) to simulate the electric field at a steady state and its static charges [16,17]. All calculations were performed using COMOL Multiphysics. The three-dimensional models were employed to simulate the electric field. The *xy*-size and height for the cis and trans chambers were 7.5 × 7.5 μm and 6.5 μm, respectively, and a 2-μm-thick Si membrane (a conductivity of 4.3 × 10^−4^ S/m and a relative permittivity (*ε*_r_) of 11.7 were used for nondoped Si [18,19]) with an IP-shaped nanopore of *D* = 2932 × 2932 nm and *d* = 100 × 100 nm was placed between the cis and trans chambers as shown in Figure 2a. In some cases, a Si_3_N_4_ layer (a conductivity of 1.0 × 10^−14^ S/m and a relative permittivity *ε*_r_ of 6.9 were used for Si_3_N_4_ [20,21]) was placed on the Si membrane. This pyramidal shape is a well-known structure because it can be fabricated by an anisotropic wet etching on a (100) silicon wafer. This chemical etching provides a slope with the angle of 54.7° due to the side (111) planes, meaning that the angle is restricted by the etching process and the crystal structure of silicon. Although it is uncertain whether 54.7° is the optimal angle for the nanopore devices, the angle was employed in this study. In the present calculations, the coordinate’s origin was located in the center of each dimension, and the topside corresponds to the ground. The bias voltage of 1.0 V was applied to the bottom terminal side, and a 137-mmol/L-phosphate-buffered saline (PBS) buffer was also employed as an electrolyte solution (Figure 2b). Compared with conventional cylindrical nanopores, the electric fields in models consisting of a 100–300-nm-thick Si_3_N_4_ with a 100-nm-diameter pore structure were also simulated (Figure A1).

## 3. Results and Discussion

### 3.1. Electric Field Gradients in Nanopore Devices

**Figure 3:**
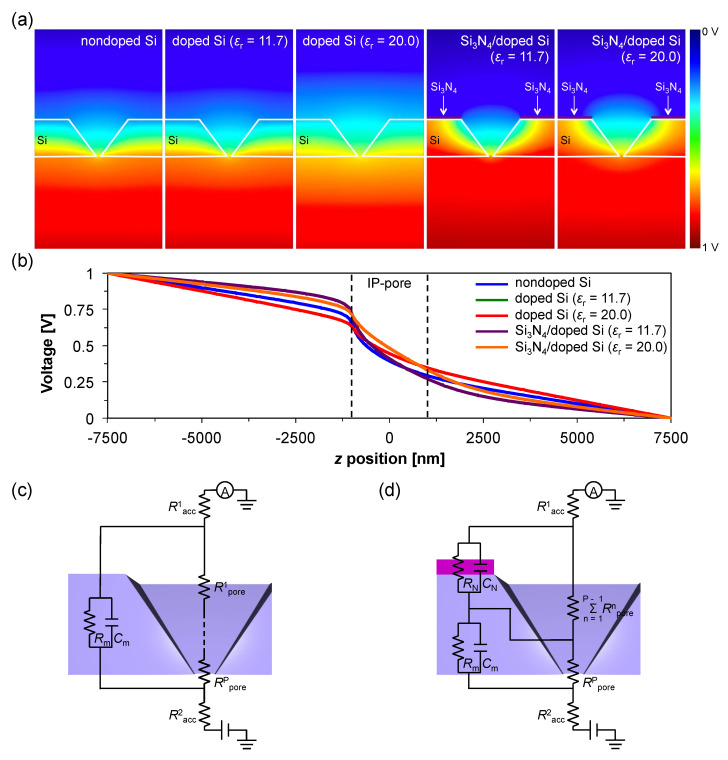
Simulation results for the IP-shaped nanopore structures. (**a**) Cross-sectional potential distributions for the nondoped Si, the doped Si, and the Si_3_N_4_/doped Si nanopores. The potential drop was found to depend on the structure and the material permittivity. (**b**) Potential dependencies on the *z* position on the center axis (*x* = 0, *y* = 0) for the nondoped Si (blue), the doped Si (*ε*_r_ = 11.7) (green), the doped Si (*ε*_r_ = 20) (red), the Si_3_N_4_/doped Si (*ε*_r_ = 11.7) (purple), and the Si_3_N_4_/doped Si (*ε*_r_ = 20) (orange) nanopores. Here, as the dependences of the nondoped Si and the doped Si (*ε*_r_ = 11.7) are almost the same property, the potential curves are overlapped. (**c**) Equivalent circuit of the Si-based IP-shaped nanopore. (**d**) Equivalent circuit of the Si_3_N_4_/Si-based IP-shaped nanopore.

For the nondoped Si-based IP-shaped nanopore structure, the potential drop is localized inside the structure (Figure 3a), however the electric field exists between the electrode and the entrance of the IP-shaped nanopore (Figure 3b (blue)). This result could be due to the large pore entrance structure, i.e., reducing the *R*_pore_, and relatively smaller resistance and larger permittivity of nondoped Si compared to those of Si_3_N_4_. Interestingly, the potential drop inside a nanopore shows a nonlinear property, as shown in Figure 3b (blue). Since the pore resistance increases with decreasing the cross-sectional area of the pore (see Equation (1)), the resistance inside the IP-shaped nanopore depends on *z*, namely, the *R*_pore_ of the structure is described as:

(3) $$R_{\mathrm{pore}}=\int_{-\frac{h}{2}}^{\frac{h}{2}}\frac{\alpha }{D{\left(z\right)}^{2}}dz$$

Thereby, the resistance corresponding to the vicinity of the nanopore exit is considerably higher than that of the vicinity of the nanopore entrance and the surface, resulting in the nonlinear potential drop. From the viewpoint of the equivalent circuit, the pore resistance of the IP-shaped nanopore is described as Σ*R*^n^_pore_ = *R*^1^_pore_ + *R*^2^_pore_ + *R*^3^_pore_ … *R*^P^_pore_, where *R*^P^_pore_ corresponds to the principal pore resistance causing the potential drop at the bottom of the nanopore (Figure 3c). The gradient of the resistance thus causes the potential curve (Figure 3b).

**Figure 4:**
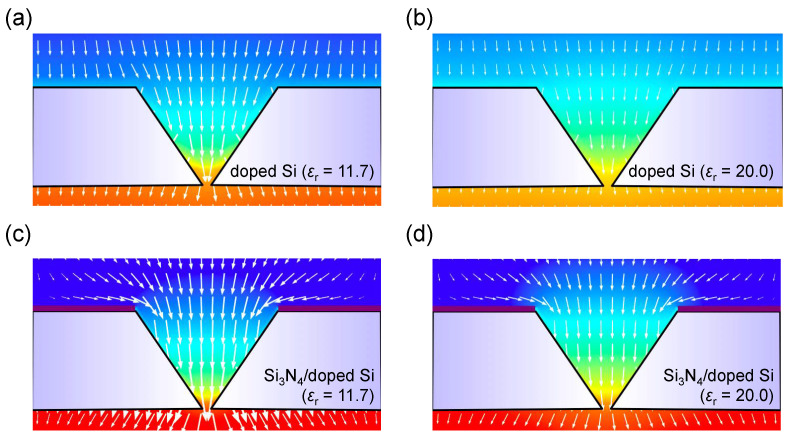
Simulation results of the vectors of the force felt by a negative charged analyte, which are the opposite directions of the electric field, in nanopore devices. (**a**) Doped Si (*ε*_r_ = 11.7). (**b**) Doped Si (*ε*_r_ = 20). (**c**) Si_3_N_4_/doped Si (*ε*_r_ = 11.7). (**d**) Si_3_N_4_/doped Si (*ε*_r_ = 20). Si_3_N_4_ layer changes the orientation of the vectors near the Si_3_N_4_ layer surface in the direction of the IP-shaped nanopore.

**Figure 5:**
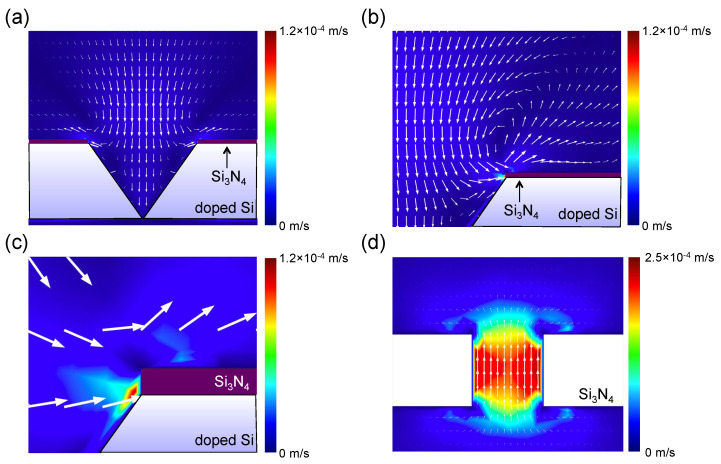
Simulated electroosmotic flows. (**a**) Electroosmotic flow velocity distribution in the Si_3_N_4_/doped Si (*ε*_r_ = 11.7) IP-shaped nanopore device. (**b**,**c**) Magnified views of the distribution in the Si_3_N_4_/doped Si IP-shaped nanopore device. (**d**) Electroosmotic flow velocity distribution in the cylindrical Si_3_N_4_ nanopore device. The electroosmotic flow in the IP-shaped nanopore seems to cause positive effects for the device throughput.

In contrast, the dispersion of the electric field in the doped Si-based IP-shaped nanopore structure shows relatively large gradients with increasing a conductivity and/or permittivity of the Si membrane, as shown in Figure 3a,b (green, red). In these instances, a conductivity of 1.0 and 10^5^ S/m, and a relative permittivity of 11.7, and 20 were used for the doped Si, respectively. These physical properties are obtained by phosphorus doping with a concentration of 4.4 × 10^13^ and 8.2 × 10^19^ cm^−3^, respectively [19,22]. Interestingly, the doped Si-based IP-shaped nanopore with *ε*_r_ = 11.7 shows almost the same potential drop to that of nondoped Si structure. This could be because the permittivity of Si is almost independent from the conductivity in the cases with less than 10^4^ S/m [19]. These homogeneous electric field gradients are suitable to drive analytes by electrophoresis. However, the vectors of the force felt by a negative charged analyte, which are opposite directions of the electric field, in a Si-based IP-shaped nanopore device are aligned along the *z*-axis (Figure 4a,b), meaning that only a small number of analytes pass through a nanopore because many of the translocated analytes along the field are ultimately captured on the membrane surface. To control the vectors, we next employed 50-nm-thick Si_3_N_4_ layers on the surface of the doped Si membranes, as shown in Figure 3a. It is interesting to note that the Si_3_N_4_ layer was found to change the vectors of the force felt by a negatively charged analyte near the Si_3_N_4_ layer surface in the direction of the IP-shaped nanopore, and the potential drops in the entire cis chamber remained, as shown in Figure 3b (purple and orange) and Figure 4c,d. These results strongly suggest that most of the analytes suspended in the cis chamber can pass through a nanopore by electrophoresis, even though analytes are suspended far away from the nanopore entrance. Indeed, the electric field exists 7500 nm above the nanopore center for each device, suggesting that the capture radius is more than 7500 nm in these device structures, which is 50 times larger than that of the conventional nanopore with *d* = 100 nm and *h* = 100 nm (Figure A1). Therefore, in a simple estimation, only taking into account the effect due to the electric field, a 12.5 × 10^4^ (=50^3^) times lower analyte concentration is the available concentration for the IP-shaped nanopore compared with the conventional nanopore. This improvement in the throughput is also comparable to that of array structures of 10^4^ nanopores per mm^2^ [11]. However, this possibility deserves careful examination because the electroosmotic flow in a device also affects the throughput [23]. Figure 5 shows simulated results of electroosmotic flow velocity distributions in a Si_3_N_4_/doped Si-based (*ε*_r_ = 11.7) IP-shaped nanopore (Figure 5a–c) and a conventional Si_3_N_4_ cylindrical nanopore (Figure 5d) device. For the IP-shaped nanopore, the electroosmotic flow does not seem to obstruct the analyte translocations from the cis to the trans chamber because the main flow is in the same direction of the vectors of the force felt by a negative charged analyte and in the perpendicular direction to the membrane (Figure 5a), whereas the Si_3_N_4_ cylindrical nanopore causes the electroosmotic flow in the opposite direction of the analyte translocations because of the negative surface charge of the Si_3_N_4_, which is a well-known behavior (Figure 5d) [24]. In the case of the IP-shaped nanopore, the electroosmotic flow in the opposite direction to that in the conventional nanopore seems to be caused by the vortical flows in the vicinity of pore entrance edges (Figure 5b). Since the Si_3_N_4_ layer on the IP-shaped Si membrane causes the electroosmotic flow in the opposite direction to analyte translocations near the Si and Si_3_N_4_ surfaces (Figure 5c), these electroosmotic flows could finally result in the vortical flows, as shown in Figure 5b. However, it is to be noted that the dynamics of the electroosmotic flow in the IP-shaped nanopore should be examined for each device because the dynamics in a conical pore structure are found to depend on the salt concentration in a buffer solution, the Debye length on the sidewall inside a nanopore, and the structure size [23,25]. It is reported that the vortical flow is generated inside a nanopore, which affects the device throughput, especially in a small pyramidal nanopore structure [23]. For the present IP-shaped nanopore device consisting of 137-mmol/L-PBS buffer, as the velocity of the electroosmotic flow is ca. 1.0 × 10^−^^5^ m/s in the vicinity of the Si_3_N_4_ surface, the analytes around the Si_3_N_4_ layer surface with *qEt*/*m* < 1.0 × 10^−^^5^ m/s cannot approach the nanopore, where *q*, *E*, *t*, and *m* denote the charge of the analytes, the electric field, time, and the mass of the analytes, respectively. Therefore, although the device throughput depends not only on the electric field and the electroosmotic flow but also the analyte physical properties, these simulation results indicate that the Si_3_N_4_/Si-based IP-shaped nanopore is one of the suitable device structures for high-throughput sensing.

**Figure 6:**
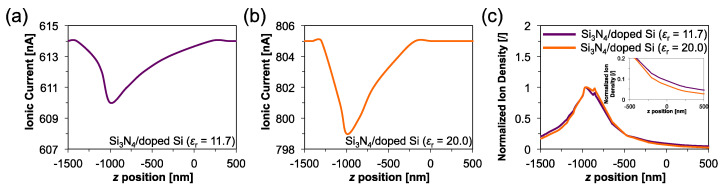
Simulated ionic current blockades. (**a**) Ionic current dependence in *z* position of a 30-nm-diameter nanoparticle in the Si_3_N_4_/doped Si (*ε*_r_ = 11.7) nanopore. (**b**) Ionic current dependence in *z* position of a 30-nm-diameter nanoparticle in the Si_3_N_4_/doped Si (*ε*_r_ = 20) nanopore. (**c**) Normalized dependences of ion density in *z* position in each nanopore device and the extended figure of the dependences in *z* = −500–500 nm (inset).

We next discuss the Si_3_N_4_/Si-based IP-shaped nanopores from the viewpoint of the ionic current blockade. Here, current blockades caused by a translocation of an insulating nanoparticle with 30-nm-diameters were simulated for the IP-shaped nanopores consisting of the Si membrane with *ε*_r_ = 11.7 and 20. As shown in Figure 6a,b, the intensities of the current blockade (*I*_p_) were found to be 4 nA and 6 nA for the nanopore structures with *ε*_r_ = 11.7 and 20, respectively, although the ratio of *I*_p_ to the base line current (*I*_b_) is almost the same for both structures. However, the widths of resistive pulses show a dependence on *ε*_r_, as show in Figure 6a,b. In the case of the nanopore with *ε*_r_ = 11.7, the ionic current is decreased by the nanoparticle suspended farther away from the bottom of the IP-shaped structure compared to the case of the nanopore with *ε*_r_ = 20. Since the ionic current blockade is caused by a nanoparticle excluding ions inside a nanopore during translocation, the difference in the widths of the current signal could arise from that the ion density in the nanopore structure with *ε*_r_ = 11.7 is distributed more broadly than that in the structure with *ε*_r_ = 20 (Figure 6c). These results indicate that the IP-shaped nanopore structures consisting of a Si_3_N_4_/Si layer can be utilized for nanopore devices as well as the conventional nanopore structures. As the structure with higher-doped Si membrane shows higher *I*_p_, a high-doped Si membrane is potentially suitable material for the IP-shaped nanopore structure when only considering the peak intensity *I*_p_.

As a qualitative explanation, the combination of the insulating layer on the semiconducting membrane and the IP-shaped nanopore structure with the large entrance plays a key role in the formation of the suitable electric field gradient within a nanopore device in terms of the high-throughput. Indeed, the electric field is localized inside a nanopore for cylindrical Si nanopore with a Si_3_N_4_ layer (Figure S1). This result would be understood using an equivalent circuit. The simplified resistance corresponding to the IP-shaped nanopore structure is equivalent to two resistances in a series circuit, i.e., Σ*R*^n^_pore_ and *R*^P^_pore_, and the Si_3_N_4_/Si layers correspond to a series of connected *RC* parallel circuits (Figure 3d). It is notable that high resistances due to Si_3_N_4_ (*R*_N_) and *R*^P^_pore_, which are placed in parallel in the other equivalent circuits and are the main factors causing the drastic potential drop, are separated from each other, as shown Figure 3d. This fact means that the drastic potential drop is separated into two steps corresponding to *R*_N_ and *R*^P^_pore_, resulting in the formation of the suitable electric field gradient within a nanopore device.

The Si_3_N_4_/Si-based IP-shaped nanopore structure is thus expected to improve the throughput of the nanopore devices. However, we should discuss the S/N ratio of the IP-shaped nanopore devices, because the large capacitance of the Si (*C*_m_) is predicted to cause greater noise in the ionic current. In the case of the Si_3_N_4_/Si-based IP-shaped nanopore, the total capacitance is estimated to be *C*_total_ = (*C*_N_ × *C*_m_)/(*C*_N_ *+ C*_m_), where *C*_N_ denotes the capacitance of the Si_3_N_4_ layer, because each capacitance corresponding to the Si_3_N_4_ and the Si is in a series circuit, meaning a smaller capacitance of the Si_3_N_4_/Si layers than that of a individual single-doped Si membrane and Si_3_N_4_ layer (Figure 3d). The noise level comparison of the Si_3_N_4_/Si-based IP-shaped nanopore and the conventional Si_3_N_4_ nanopore is experimentally examined in the next section.

### 3.2. Fabrications and Ionic Current Noise Level of the IP-Shaped Nanopore Devices

**Figure 7:**
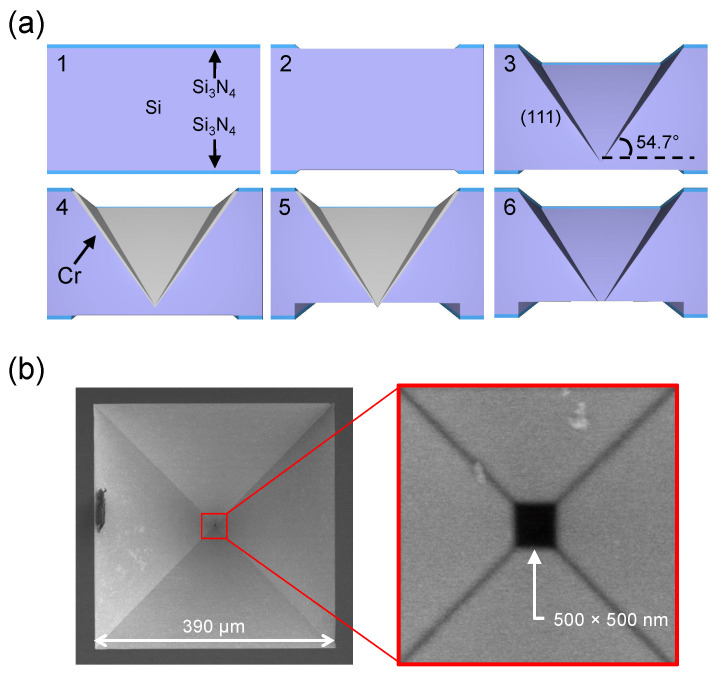
Fabrication process for the Si_3_N_4_/Si-based IP-shaped nanopore structures and scanning electron microscope (SEM) images of the nanostructures. (**a**) Schematic illustrations of the fabrication process. The two-step etching processes (No. 3 and 5) enabled us to fabricate the nanostructures without electron beam lithography. (**b**) SEM images of the Si_3_N_4_/Si-based IP-shaped nanopore structures with *D*/*d* = ca. 1000 fabricated using the present method.

**Figure A2:**
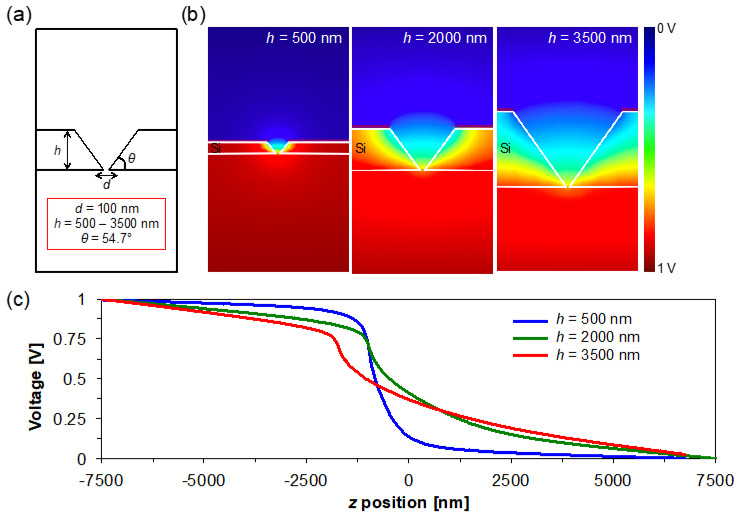
Dependence of the electric field distribution on the thickness of Si membrane for the Si_3_N_4_/Si-based nanopore structures. (**a**) Schematic illustration of the simulation model. (**b**) Cross-sectional potential distributions for the nanopores with *h* = 500 nm, 2000 nm, and 3500 nm. It was found that higher thickness causes more gradual potential drop in a nanopore device. (**c**) Potential dependences on *z* position on the center axis (*x* = 0, *y* = 0) for the nanopores with *h* = 500 nm (blue), 2000 nm (green), and 3500 nm (red).

Finally, we discuss fabrications and the ionic current noise level of the IP-shaped nanopore that facilitate high-throughput nanopore devices. As mentioned above, the IP-shape with *θ* = 54.7° can be fabricated by an anisotropic wet etching on a (100) silicon wafer. However, controlling the nanopore size (*d*) is difficult using this method because the thickness of Si wafer usually has a margin of error of about ± ca.10%. Si wafers with a nanometer thickness are thus preferred for the nanopore fabrications with an accuracy of a couple of nanometers. However, the higher thickness of the Si membrane (*h*) is a more appropriate structure to improve the device throughput, because a higher thickness causes a more gradual potential drop in a nanopore device (Figure A2). This fact means that the difficulty of the fabrication increases with *h* due to the increasing error thickness with *h*. Hence, the Si-based IP-shaped nanopore structures with micrometer thicknesses have not been reported, though structures with nanometer thicknesses are well-known [26,27]. Indeed, S. Zeng et al. recently report pyramidal nanopore structures with a ca. 80 nm thickness [23]. To fabricate the IP-shaped nanopore structures with micrometer thicknesses, we employed a two-step etching process in this study (Figure 7a).

In this study, 300-μm-thick Si wafers, both sides of which were covered with 50-nm-thick Si_3_N_4_ membranes, were employed as a substrate. First, 390-μm-square and 1.0-mm-square windows on the top and the bottom sides of the Si_3_N_4_ membrane were respectively fabricated by photolithography (Figure 7a-2). The topside Si_3_N_4_ window was wet-etched in a KOH solution at 120 °C for 60 min (step I), resulting in a 275.4-μm-height IP-shape (Figure 7a-3). After that, a 20-nm-thick Cr film was deposited as a protection film by sputtering on the etched area (Figure 7a-4), and subsequently the bottom side window was wet-etched in a KOH solution at 30 °C with a slow etching rate (step II), and the etching went so far as to reach the Cr film (Figure 7a-5). Here, reaching the Cr film was confirmed by an alternating scanning electron microscope (SEM) observation and the slow KOH etching. Finally, the Si_3_N_4_/Si-based IP-shaped nanopore structure with *D* = 390 μm and *h* = 275 μm was obtained by removing the Cr film, as shown in Figure 7b. Because the two-step etching process enabled us to fabricate the nanopore structures without electron beam lithography, the present fabrication method is preferable from the perspective of fabrication costs. However, the fabrication of a square-shaped nanopore with *d* = 100 nm was found to be difficult using this method because the etching rate is not exactly the same among each (111) surface of silicon. These experimental errors of the etching rate result in a rectangular-shaped nanopore with a width of 100 nm. Thus, a nanopore with *d* = 500 nm is currently the minimum square-shaped structure using this method. However, since the electric field properties of the IP-shaped nanopore are almost independent from the pore size, the 500-nm IP-shaped nanopore would also demonstrate high-throughput sensing (Figure S2).

**Figure 8:**
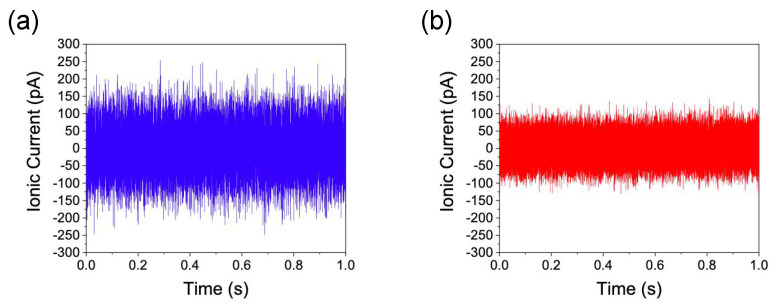
Noise level of the ionic currents. (**a**) Ionic current in a conventional Si_3_N_4_ nanopore. (**b**) Ionic current in a Si_3_N_4_/doped Si-based IP-shaped nanopore, i.e., doped Si-based IP-shaped nanopore with a Si_3_N_4_ layer. Here, the central value of each current is normalized at 0 A. It was found that peak-to-peak noise level of the IP-shaped nanopore is lower than that of the conventional cylindrical Si_3_N_4_ nanopore.

Figure 8 shows the noise level comparison of the Si_3_N_4_/Si-based IP-shaped nanopore and the conventional cylindrical Si_3_N_4_ nanopore. Here, a phosphorus-doped Si wafer with 5 S/m and 137-mmol/L-PBS buffer were employed as a Si membrane and buffer solution, respectively, and an electrophoretic field of 100 mV was applied to the pore, utilizing two Ag/AgCl electrodes. The ionic current was detected at 1 MHz by employing a home-built current amplifier backed by a digitizer (NI-5922, National Instruments, Austin, TX, USA) and stored in a RAID hard drive (HDD-8265, National Instruments, Austin, TX, USA) under LabVIEW control [7]. Thereafter, 1 MHz ionic current–time data were finally compressed to 100 kHz and normalized at 0 A to compare the peak-to-peak noise level between the two ionic current data. As shown in Figure 8, the peak-to-peak level of ionic current for the IP-shaped nanopores is lower than that of conventional nanopores. This result indicates that the total capacitance of the IP-shaped nanopore structure *C*_total_ is smaller than that of conventional Si_3_N_4_ nanopore structure *C*_N_. Indeed, *C*_total_ = (*C*_N_ × *C*_m_)/(*C*_N_ *+ C*_m_) and *C*_N_ are estimated to be *C*_total_ = 2.16 × 10^−10^ F and *C*_N_ = 7.64 × 10^−7^ F, respectively, as *μ*_N_ = 6.109 × 10^−11^ F/m, *S*_N_ = 6.25 × 10^−4^ m^2^, *h*_N_ = 5.0 × 10^−8^ m, *C*_m_ = 2.158 × 10^−10^ F, *μ*_m_ = 1.036 × 10^−10^ F/m, *S*_m_ = 6.25 × 10^−4^ m^2^, *h*_m_ = 3.00 × 10^−4^ m. Here, *μ*_N_, *μ*_m_, *S*_N_, *S*_m_, *h*_N_, and *h*_m_ correspond to the permittivity, the area, and the thickness of Si_3_N_4_ layer (N) and Si membrane (m), respectively.

## 4. Conclusions

Si_3_N_4_/Si-based IP-shaped nanopore structures, which could be fabricated only by photolithography, were proposed as structures for high-throughput nanopore devices. The insulating Si_3_N_4_ layer on the doped Si membrane and the large entrance of the IP-shaped nanopore structure were found to cause a three-dimensionally homogeneous potential drop in the entire cis chamber, and the electric field distributed in the direction of the nanopore entrance in the vicinity of the Si_3_N_4_ layer surface. These modifications of the electric field increase the capture radius for analytes, suggesting higher throughput for the presented devices. The homogeneous potential drop within the IP-shaped nanopore could provide another advantage from the perspective of the sensing accuracy of the nanopore devices because the homogeneous potential drop causes slower translocation speed of analytes compared with the drastic potential drop in the conventional cylindrical nanopores. In some cases of nanopore devices, since the translocation speed is too fast to analyze single molecules and/or single bioparticles passing through a nanopore, reducing the analyte translocation speed is the major challenge [2,7,28]. Thus, reducing the translocation speed due to the homogeneous potential drop inside the IP-shaped nanopore is expected to improve the sensing accuracy, such as spatial resolution in the shape analysis [7]. However, it is to be noted that the sensing accuracy also depends on the ratio of signal to noise of the ionic current and the shape of nanopore. In addition, the nanopore sensing is currently limited to relatively large molecules, such as long DNA and large proteins, and single bioparticles, such as viruses. Further improvement of the sensing accuracy for the identification of small, single molecules is a subject for future study. The present results suggest a high potential for the Si_3_N_4_/Si-based IP-shaped nanopore to serve as a novel device structure that facilitates high-throughput sensing for single molecules and bioparticles; additionally, the present findings present new methods for controlling the electric field in nanofluidics.

## Supplementary Materials

The following are available online at https://www.mdpi.com/2072-666X/11/10/893/s1, Figure S1: Electric field in the Si_3_N_4_/Si cylindrical nanopore, Figure S2: Electrical field in the Si_3_N_4_/Si IP-shaped nanopore with *d* = 500 nm.
