# Supplementary material for: Pore Structures for High-Throughput Nanopore Devices
Source: Micromachines (Basel). 2020 Sep 26;11(10):893. doi: 10.3390/mi11100893 (PMC7600762; doi:10.3390/mi11100893)
Supplement: Supplementary file 1 [file micromachines-11-00893-s001.pdf]

Supplementary Materials for

Pore structures for high-throughput nanopore devices

The supplementary Information includes Supporting Figures (Figs. S1-S2)

SI 1. Electric field in the Si_3N_4/Si cylindrical nanopore.

SI 2. Electrical field in the Si_3N_4/Si IP-shaped nanopore with *d* = 500nm.

SI 1. Electric field in the Si_3N_4/Si cylindrical nanopore.

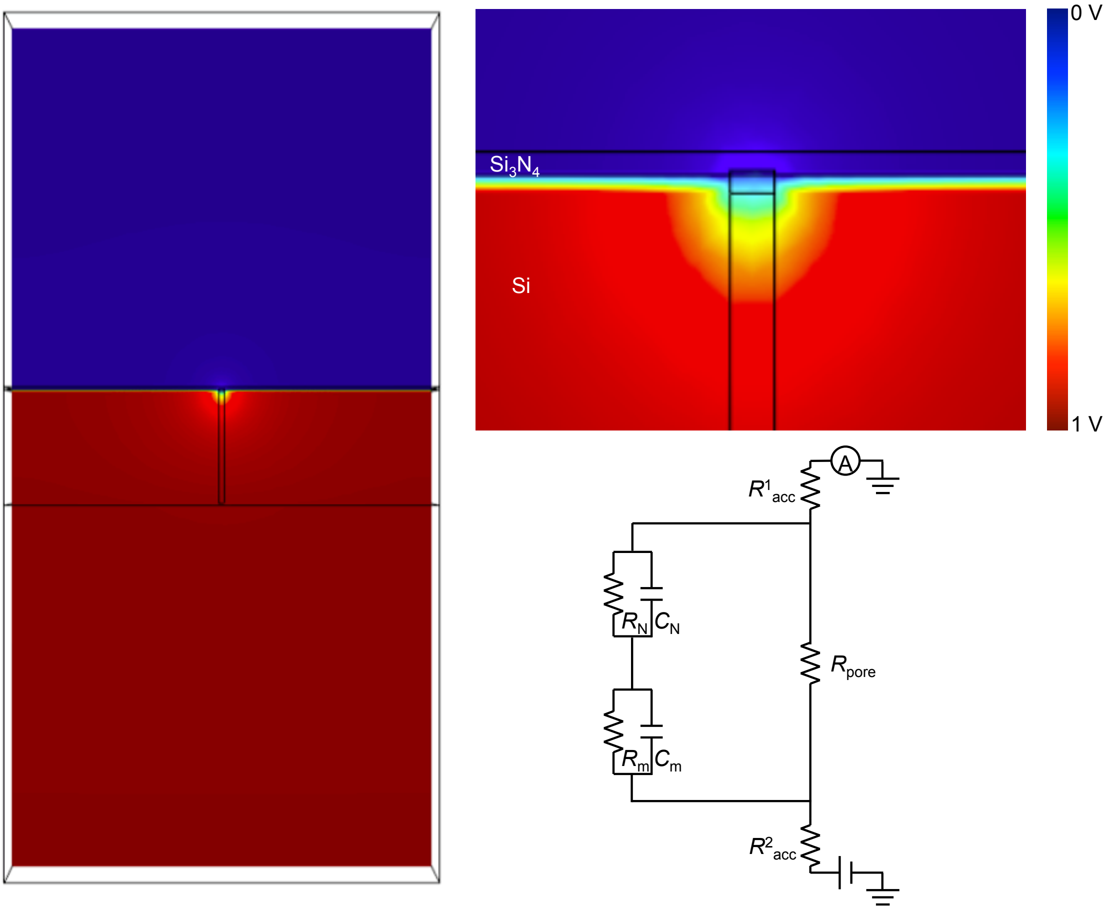

Figure S1. Electric field in the Si_3N_4/Si cylindrical nanopore.

As shown in Figure S1, this structure cannot cause the suitable electric field gradient within a nanopore device because the resistance of Si_3N_4 layer (R_N) and the pore resistance (R_{pore}) are placed in a parallel in the equivalent circuits for this structure. This equivalent circuit is essentially the same to that of the conventional cylinder nanopores.

SI 2. Electrical field in the Si_3N_4/Si IP-shaped nanopore with *d* = 500nm.

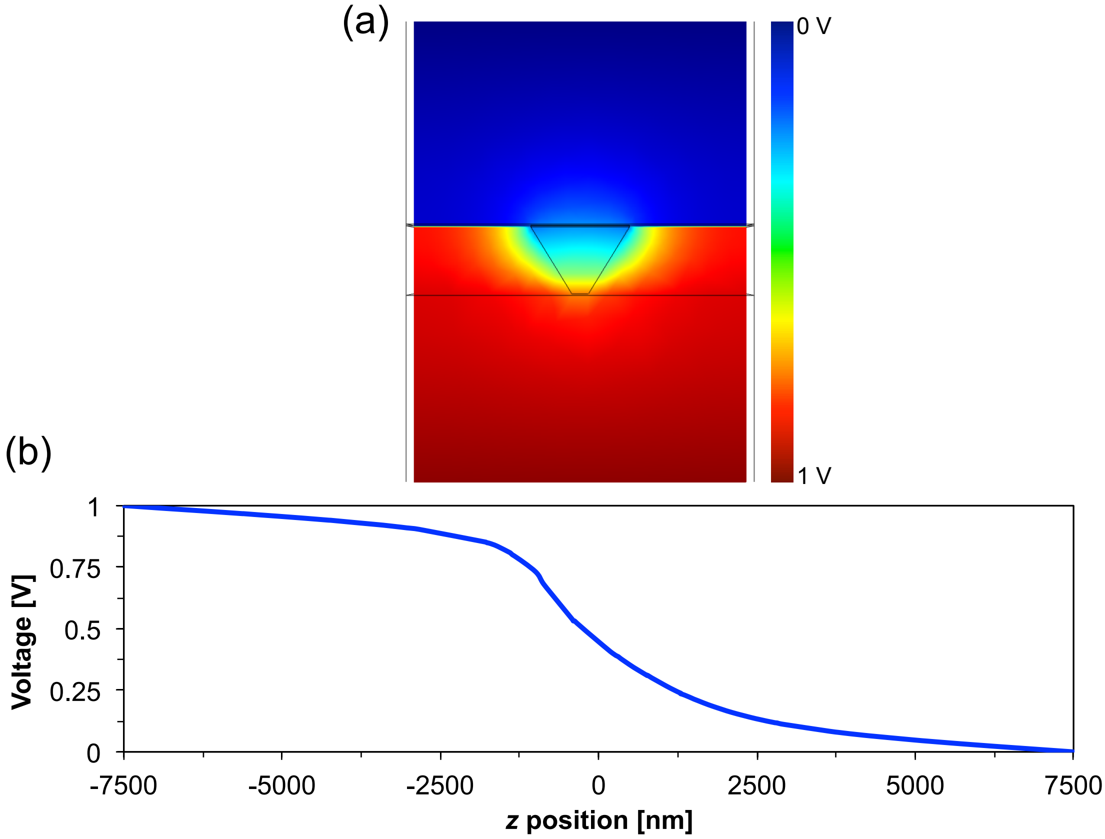

Figure S2. Electrical field in the Si3N4/Si IP-shaped nanopore with *d* = 500nm.

As shown in Figure S2, the Si_3N_4/Si IP-shaped nanopore with *d* = 500 nm results in almost the same electric field to that in the Si_3N_4/Si IP-shaped nanopore with *d* = 100 nm, meaning that the 500-nm-IP-shaped nanopore could show a high-throughput sensing as well as the IP-shaped nanopore discussed in the main text.
